# Local Lung HIF-1α and VEGF Activation to Reverse Emphysema by a Sulfated Caffeic Acid Dehydropolymer

**DOI:** 10.3390/biology15070564

**Published:** 2026-04-01

**Authors:** Tien M. Truong, Meghan L. Thompson, Umesh R. Desai, Masahiro Sakagami

**Affiliations:** 1 Department of Pharmaceutics, School of Pharmacy, Virginia Commonwealth University, 410 North 12th Street, Richmond, VA 23298, USA; 2 Department of Medicinal Chemistry, School of Pharmacy, Center for Drug Discovery, Virginia Commonwealth University, 800 East Leigh Street, Richmond, VA 23219, USA

**Keywords:** emphysema, caffeic acid, dehydropolymer, hypoxia-inducible factor (HIF), vascular endothelial growth factor (VEGF), reversal

## Abstract

In emphysema, the alveolar septal structure is progressively destroyed, which is believed to be irreversible. However, as it has recently been linked to vascular endothelial growth factor (VEGF) deficiency, we hypothesized that VEGF stimulation can promote lung cell proliferation/migration to reverse emphysema. Our sulfated caffeic acid dehydropolymer, CDSO3, was thus examined in vitro and in vivo, given its VEGF-stimulating activity via ferrous ion (Fe^2+^) chelation-mediated stabilization of hypoxia-inducible factor-1α (HIF-1α). In lung epithelial/endothelial cells, CDSO3 promoted proliferation and wound closure by 1.6–3.0-fold at 10 μM; however, these effects were negated by excess FeSO_4_ or an HIF-1α inhibitor, indicating an Fe^2+^- and HIF-1α-dependent mechanism. In rat models of *established* emphysema induced by cigarette smoke extract or the VEGF receptor antagonist SU5416, two-week lung administration of CDSO3 at 60 μg/kg from day 21 enabled: 68–79% recovery of exercise endurance and airspace enlargement/destruction; a 1.8-fold increase in proliferating cell nuclear antigen above healthy levels; normalization of cleaved caspase-3; restoration of HIF-1α; and a 1.3-fold increase in VEGF above healthy levels. In contrast, CDSO3 pre-chelated with Fe^2+^ was ineffective. In conclusion, Fe^2+^ chelation-mediated HIF-1α stabilization and VEGF stimulation via local lung delivery of CDSO3 can reverse *established* emphysema by promoting cell growth and survival.

## Simple Summary

Emphysema is a serious lung disease in which alveolar walls gradually and irreversibly break down, eventually leading to death without a cure. However, as this lung tissue destruction has recently been linked to a deficiency in the lung’s vascular endothelial growth factor (VEGF), we investigated whether our compound, CDSO3 (sulfated caffeic acid dehydropolymer), could help repair emphysema using lung cells and disease rat models. CDSO3 increases VEGF by chelating ferrous ions (Fe^2+^), which stabilizes the upstream regulator hypoxia-inducible factor-1α (HIF-1α). In lung cells, CDSO3 potently promoted cell growth and wound closure; however, these effects disappeared when excess Fe^2+^ or an HIF-1α inhibitor was added, indicating that CDSO3 acts through these pathways. In rats with *established* emphysema induced by cigarette smoke extract or VEGF receptor blockade, two-week local lung delivery led to marked improvements: rats regained ~70% of lost exercise capacity, lung structure improved, lung cells showed renewed growth, cell death decreased, HIF-1α levels were restored, and VEGF levels exceeded those of healthy lungs. In contrast, CDSO3 pre-chelated with Fe^2+^ was no longer effective. These findings suggest that activating HIF-1α and VEGF locally in the lung via CDSO3 delivery can reverse *established* emphysema by promoting lung cell growth and survival.

## 1. Introduction

With cigarette smoking as its primary risk factor, emphysema is classified as a chronic lung injury and clinically manifests in chronic obstructive pulmonary disease (COPD) and alpha-1 antitrypsin deficiency (AATD) [1,2]. This fatal lung disease is characterized by the progressive destruction and loss of alveolar septal walls and structures [1,2]. The resulting decline in breathing ability and lung function leads to physical and functional disability, respiratory failure, and ultimately death [1,2]. Therefore, to better understand and treat emphysema, several pathobiological mechanisms have been proposed and are being investigated, including induced proteolysis, oxidative stress, chronic inflammation, and more recently, vascular endothelial growth factor (VEGF) deficiency [3,4,5]. Despite these efforts, however, alveolar structural destruction and loss in emphysema are still considered irreversible and incurable. To date, no drug can reverse emphysema in patients, and bronchodilators and corticosteroids remain the main treatment options, offering only palliative relief of symptoms [6].

**Figure 1:**
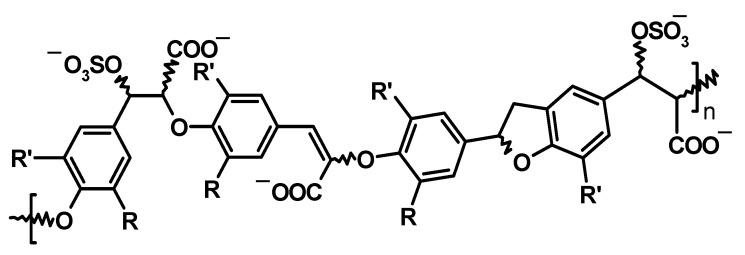
Sulfated caffeic acid dehydropolymer, CDSO3. R = OH^−^, R’ = H, n = 5–13; sulfated group per caffeic acid monomer = 0.04; weight-average molecular weight = 3320 g/mol [7].

Our sulfated caffeic acid dehydropolymer, CDSO3 (Figure 1), was originally designed as a low-molecular-weight lignin-like oligomer and found as a novel proprietary inhibitor of serine proteases, including factor Xa and thrombin [7]. We have then previously shown that CDSO3 could represent a new class of anti-emphysema drug for local lung delivery due to its unique and potent multifunctional [anti-elastase, anti-oxidative, and anti-inflammatory] activities at low micromolar (μM) concentration levels [8]. Accordingly, CDSO3 protected against in vitro lung cell death induced by various emphysematous insults and prevented and intervened in the development of experimental emphysema in rats when administered locally to the lungs [9,10]. Even so, the anti-cell death activities alone did not indicate that CDSO3 could promote repair and reconstruction of damaged lungs in emphysema, although such outcomes are clinically more desired.

In this context, we further identified that CDSO3 potently chelated ferrous ion (Fe^2+^), stabilized hypoxia-inducible factor-1α (HIF-1α), an upstream transcription factor of VEGF, and stimulated VEGF in the lungs of healthy rats following local lung administration [10]. Because such HIF-1α and VEGF activation in the lung is expected to facilitate the growth of new lung cells, this study first examined whether CDSO3 promotes proliferation and migration in in vitro lung cell models. The mechanistic involvement of Fe^2+^ chelation and HIF-1α was also assessed using excess FeSO_4_ and the HIF-1α inhibitor CAY10585. CDSO3 was then tested for its ability to reverse established emphysema in two in vivo rat models induced by cigarette smoke extract (CSE) and the VEGF receptor antagonist SU5416. CDSO3 pre-mixed with FeSO_4_ was also administered to these emphysematous rats to evaluate the role of Fe^2+^ chelation for mechanistic validation.

## 2. Materials and Methods

### 2.1. CDSO3, Sulfated Caffeic Acid Dehydropolymer (Figure 1)

CDSO3 is a sulfated dehydropolymer of caffeic acid, which was chemoenzymatically synthesized from caffeic acid as previously described [7]. This proprietary molecule is a 5–13-mer, predominantly featuring β-5 and β-*O*-4 intermonomer linkages, with an average of 0.4 sulfates/monomer, resulting in a weight-average molecular weight of 3320 g/mol [7]. CDSO3 was stored at −20 °C in concentrated aqueous aliquots, from which test and dosing solutions were freshly prepared for each experiment.

### 2.2. In Vitro Lung Cell Proliferation and Migration Activities

Human alveolar epithelial A549 cells (American Type Culture Collection, Manassas, VA, USA) and human lung microvascular endothelial (HMVEC-L) cells (Lonza, Walkersville, MD, USA) were used to assess proliferation and migration. Non-confluent A549 and HMVEC-L cells (40,000 cells/well, 96-well plates) were treated with CDSO3 at 0–10 μM in serum-suppressed (1%) media (SSM) for 48 h and in growth media (GM; Lonza) for 24 h, respectively. Cell proliferation was measured using BrdU [5-bromo-2-deoxyuridine] (450 nm; BioVision, Milpitas, CA, USA) and MTT [3-(4,5-dimethylthiazol-2-yl)-2,5-diphenyltetrazolium bromide] (570 nm; Cayman Chemical, Ann Arbor, MI, USA) assays, as per the manufacturers’ protocols. Cell migration was assessed by the scratch wound closure assay. Confluent cell monolayers in 6-well plates were scratched with a pipette tip and then treated with CDSO3 at 0–10 μM in SSM for 48 h and GM for 24 h. The % cell wound closure was calculated from: (A_pre_ − A_post_)/A_pre_ × 100, where A_pre_ and A_post_ were the sums of the scratch wound areas at three different locations in a well before and after treatment, respectively, analyzed using ImageJ 1.53c (National Institute of Health, Bethesda, MD, USA). In both studies, CDSO3 at 10 μM was also tested (1) after pre-mixing with 50 μM FeSO_4_ (Sigma-Aldrich, St. Louis, MO, USA) for 15 min before addition to the cells, and (2) in cells pre- and co-treated with 5 μM CAY10585 (HIF-1α inhibitor; Cayman Chemical) from 1 h before CDSO3 addition, to evaluate the mechanistic involvement of Fe^2+^ chelation and HIF-1α, respectively.

### 2.3. In Vivo Reversal Activities in Two Rat Models of Established Emphysema

CDSO3 was tested for its in vivo reversal activities against established emphysema in two rat models induced by CSE and SU5416. The animal study protocol was approved by the Institutional Animal Care and Use Committee of Virginia Commonwealth University. Adult male Sprague-Dawley rats (~250 g body weight; Hilltop Lab Animals, Scottdale, PA, USA) were housed in a vivarium maintained at 20–23 °C, 40–70% relative humidity, and 12 h light/dark cycles with free access to food and water. After 3 days of acclimatization, rats were trained to run on an AccuPacer rodent treadmill (Accuscan Instruments, Columbus, OH, USA) throughout the study, as established in-house [9,10,11].

CSE- and SU5416-induced emphysema was developed and established as reported previously [10,11]. In the CSE-induced model, rats received CSE via three intraperitoneal injections on days 1, 8, and 15 and were left untreated until day 21. The CSE solution for injection was freshly prepared for each rat by bubbling mainstream smoke from a 3R4F research cigarette (University of Kentucky, Lexington, KY, USA) through 1 mL of saline over ice using a custom-built smoking apparatus. In the SU5416-induced model, rats received SU5416 (Cayman Chemical) at 20 mg/kg in suspension via subcutaneous injection and were left untreated until day 21. In both models, treadmill exercise endurance times on day 21 were significantly reduced to 4–16 min, compared to ~45 min for healthy rats, confirming the development of established emphysema.

From day 21, rats in each emphysema model received orotracheal (OT) spray instillations (0.1 mL) of (1) saline; (2) CDSO3 at 10 μg/kg; (3) CDSO3 at 60 μg/kg; or (4) CDSO3 at 60 μg/kg pre-mixed with 10 mM FeSO_4_, three times weekly for two weeks until day 35. Two additional groups were normal healthy rats without emphysema induction, left untreated or OT-dosed with CDSO3 at 60 μg/kg three times weekly for two weeks. OT spray instillations were performed using a MicroSprayer (PennCentury, Wyndmoor, PA, USA) under brief (4 min) anesthesia with 4% isoflurane [10,11]. On day 35, post-treatment exercise endurance times were measured on the treadmill, followed by euthanasia under anesthesia with intraperitoneal urethane (1 g/kg; Sigma-Aldrich) and lung harvest. The left lungs were inflated with ~8 mL of 45 °C agarose solution (0.5%; Sigma-Aldrich) via a tracheal cannula at a hydrostatic pressure of 20 cm, placed on ice to solidify the agarose, and fixed in 10% phosphate-buffered formalin (Thermo Fisher Scientific, Waltham, MA, USA) at 4 °C for morphological airspace assessments described below. Before formalin fixation, the right lung lobes were removed and snap-frozen in liquid nitrogen for several biomarker analyses, also described below.

### 2.4. Morphological Assessments of Airspace Enlargement and Destruction

Mean linear intercept (MLI) and % destructive index (%DI) were determined as previously described [9,10,11] to assess morphological changes in the alveolar airspaces. The MLI was calculated as the average alveolus size for each rat from five randomly selected hematoxylin/eosin-stained alveolar airspace images (3450 × 2585 μm) taken from formalin-fixed, agarose-inflated left lungs. In each image, five equally spaced horizontal lines were drawn, their intersections with alveolar walls were counted, and the MLI was calculated from: MLI = 17,250 (3450 × 5) μm/total number of intersections. The %DI was obtained by counting normal or destructed alveoli in 50 non-overlapping alveolar images captured at 100× magnification. A grid of 66 equally spaced dot points was overlaid on each image, and the alveolus surrounding each dot point was evaluated according to the criteria of Eidelman et al. [12]. For each rat, a minimum of 2000 dot points were assessed. The %DI was calculated from: %DI = D/(D + N) × 100, where D and N represent the numbers of destructed and normal alveoli, respectively.

### 2.5. Immunohistochemical Assessment of Alveolar PCNA Expression

Immunohistochemical expression of proliferating cell nuclear antigen (PCNA), a marker of cell proliferation, in the lungs was assessed using unstained alveolar sections from the left lungs. The sections were deparaffinized in xylene and rehydrated through graded alcohols. Heat-mediated antigen retrieval was performed in citrate buffer (pH 6.0) at 95 °C for 15 min. Endogenous peroxidase activity was quenched with 3% hydrogen peroxide for 10 min, and non-specific binding was blocked with 5% horse serum for 30 min at room temperature. The sections were then incubated with anti-PCNA antibody (1:200; Cell Signaling Technology, Danvers, MA, USA) at 4 °C for 24 h, followed by incubation with horseradish peroxidase (HRP)-conjugated anti-immunoglobulin G (IgG) antibody for 30 min at room temperature. PCNA was visualized using DAB [3,3′-diaminobenzidine] for 3 min, and the alveolar septa were counterstained with hematoxylin. Unless otherwise specified, all chemicals and reagents used above were obtained from Sigma-Aldrich.

### 2.6. Western Blot Analysis

Lung tissue expression levels of the proteins of interest were measured by Western blotting, as described previously [10,11]. Briefly, tissue lysates from the right lung lobes were prepared in the presence of protease inhibitor cocktails. Protein samples (40 μg) were denatured, electrophoresed, and transferred to membranes. The membranes were incubated with primary antibodies against: (1) PCNA (1:2000; Cell Signaling Technology); (2) cleaved caspase-3 (cCasp3; 1:500; Cell Signaling Technology); (3) HIF-1α (1:1000; Thermo Fisher Scientific); and (4) VEGF (1:100; Santa Cruz Biotechnology, Dallas, TX, USA). Target proteins were detected using an HRP-conjugated anti-IgG antibody, followed by reaction with SuperSignal West Pico chemiluminescent substrate (Pierce, Rockford, IL, USA). After overnight washing, the membranes were re-probed with anti-β-actin antibody (1:5000; Sigma-Aldrich) and processed similarly to detect β-actin as loading controls. Bands of interest were visualized using an X-Omat 2000A processor (Eastman Kodak, Rochester, NY, USA) and quantified with ImageJ. The intensity of each target protein was normalized to the β-actin signal and expressed relative to that in healthy rat lung samples.

### 2.7. Data Description and Statistical Analysis

In vitro lung cell studies were conducted with n = 3 per treatment. In vivo animal studies included n = 5–9 per group, while Western blot analyses were performed using representative samples of n = 3 or 5. Individual animal data are presented together with group mean ± standard deviation (SD). Statistical differences among treatment groups were assessed by analysis of variance (ANOVA), followed by Tukey’s multiple comparison test using JMP Pro 14 (SAS Institute, Cary, NC, USA). Changes in exercise endurance time within each treatment group were compared with 0 using Student’s *t*-test. For all analyses, *p* < 0.05 was considered statistically significant.

## 3. Results

### 3.1. Stimulation of In Vitro Lung Cell Proliferation

**Figure 2:**
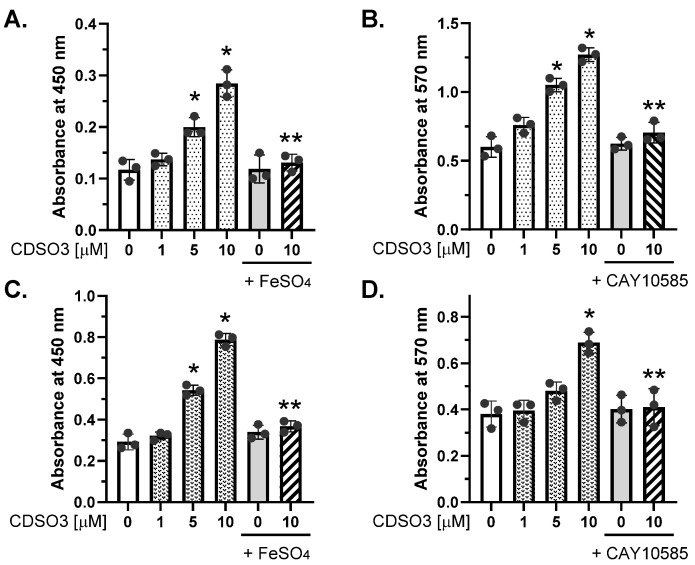
In vitro stimulatory activities of CDSO3 at 0–10 μM on (**A**,**B**) A549 and (**C**,**D**) HMVEC-L cell proliferation, measured by absorbance at 450 nm (BrdU assay) and 570 nm (MTT assay), respectively. CDSO3 at 10 μM was also tested after pre-mixing with 50 μM FeSO4 or with the cells pre/co-incubated with 5 μM CAY10585 (HIF-1α inhibitor). Data: mean ± SD (n = 3). Dots: individual well data. * *p* < 0.05 vs. vehicle control; ** *p* < 0.05 vs. CDSO3 at 10 μM.

CDSO3 potently stimulated proliferation of lung epithelial (A549) and endothelial (HMVEC-L) cells in a concentration-dependent manner, achieving 2–3-fold increases at 10 μM, as shown in Figure 2A,B and Figure 2C,D, respectively, measured by BrdU and MTT assays. However, when CDSO3 was pre-mixed with excess Fe^2+^ (FeSO_4_), the stimulatory effect was abolished, whereas FeSO_4_ alone had no effect on proliferation (Figure 2A,C). CDSO3 also failed to stimulate cell proliferation when cells were pre/co-incubated with CAY10585, while the HIF-1α inhibitor alone did not affect proliferation (Figure 2B,D). These results indicate that CDSO3’s stimulatory activity of lung cell proliferation involves Fe^2+^ chelation and HIF-1α as a mechanism.

### 3.2. Promotion of In Vitro Lung Cell Wound Closure

**Figure 3:**
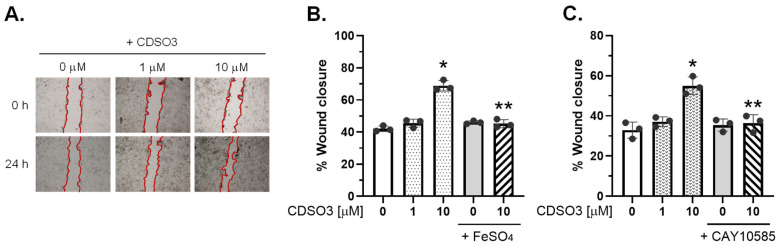
In vitro promoting activities of CDSO3 at 0–10 μM on (**A**,**B**) A549 and (**C**) HMVEC-L cell migration, assessed using a scratch wound closure assay. (**A**) Representative cell images before and after treatment; and (**B**,**C**) % wound closure under various test conditions. CDSO3 at 10 μM was also tested after pre-mixing with 50 μM FeSO_4_ or with the cells pre/co-incubated with 5 μM CAY10585 (HIF-1α inhibitor). Data: mean ± SD (n = 3). Dots: individual well data. * *p* < 0.05 vs. vehicle control; ** *p* < 0.05 vs. CDSO3 at 10 μM.

CDSO3 potently promoted proliferative and migratory wound closure in A549 (Figure 3A,B) and HMVEC-L (Figure 3C) cells in a concentration-related manner, achieving a significant 1.6-fold increase at 10 μM in both cell types. When pre-mixed with excess Fe^2+^ (FeSO_4_), however, CDSO3 was unable to promote wound closure, whereas FeSO_4_ alone had no effect (Figure 3B). CDSO3 also did not promote wound closure when cells were pre/co-incubated with CAY10585, while the HIF-1α inhibitor alone had no impact (Figure 3C). These results again indicate that CDSO3’s promoting activity of lung cell proliferative and migratory wound closure is mediated via Fe^2+^ chelation and HIF-1α.

### 3.3. Recovery of Exercise Endurance in Rats with Established Emphysema

**Figure 4:**
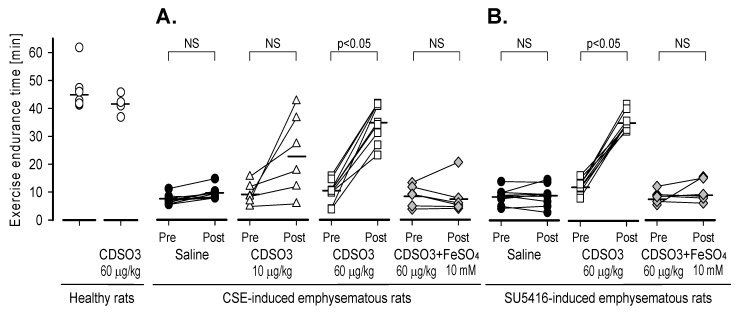
Pre- and post-treatment treadmill exercise endurance times in (**A**) CSE- and (**B**) SU5416-induced emphysematous rats treated with saline or CDSO3 at 10 or 60 μg/kg, compared with healthy rats. CDSO3 at 60 μg/kg was also tested after pre-mixing with 10 mM FeSO_4_. On day 21, exercise endurance times significantly declined to 4–16 min in emphysema-induced animals. CDSO3 or saline was then administered to the lungs three times weekly for two weeks until day 35. Treatment group: n = 9, 6 or 5. Data: individual animal values. Horizontal bars: group means. *p* < 0.05 vs. no change (i.e., 0); NS: not significant.

Two-week treatment of CDSO3 markedly improved treadmill exercise endurance in both (A) CSE- and (B) SU5416-induced emphysematous rats in a dose-related manner, with significant recovery observed at 60 μg/kg in both models (Figure 4). On day 21, pre-treatment exercise endurance times were only 4–16 min in both emphysema models, which was a 64–92% reduction from 45.6 ± 6.5 min in healthy controls, confirming established emphysema. In these animals, saline treatment did not improve endurance (3–15 min on day 35). In contrast, after CDSO3 (60 μg/kg) treatment, exercise endurance recovered by 71% to 35.2 ± 7.0 min in the CSE-induced model and by 70% to 35.6 ± 3.3 min in the SU5416-induced model. However, CDSO3 pre-mixed with FeSO_4_ did not improve exercise endurance in either model, suggesting a mechanism mediated by Fe^2+^ chelation. In healthy rats, CDSO3 (60 μg/kg) had no effect on running performance.

### 3.4. Alveolar Structural Recovery in Rats with Established Emphysema

**Figure 5:**
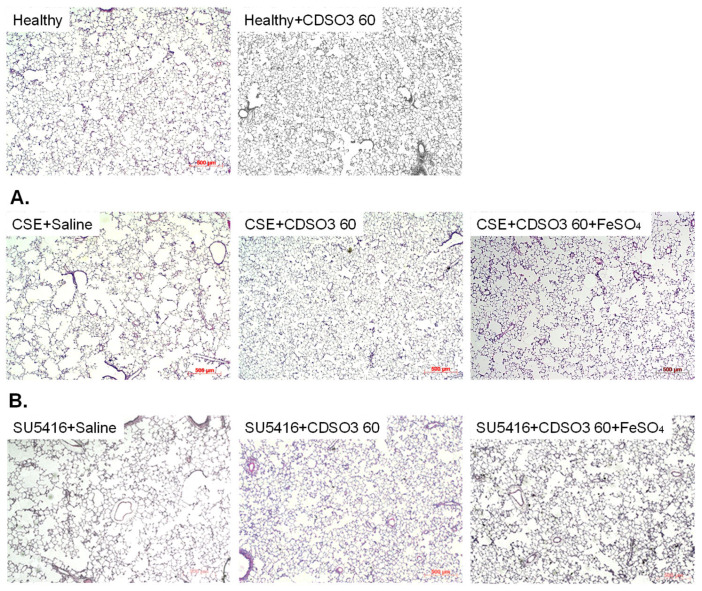
Representative images of alveolar airspaces for (**A**) CSE- and (**B**) SU5416-induced emphysematous rats treated with saline or CDSO3 at 10 or 60 μg/kg, compared with healthy rats (scale bar = 500 μm). CDSO3 at 60 μg/kg was also tested after pre-mixing with 10 mM FeSO_4_.

CDSO3 treatment also effectively restored alveolar airspace structure in both (A) CSE- and (B) SU5416-induced emphysematous rats, with the greatest recovery observed at 60 μg/kg (Figure 5). MLI and %DI values for each treatment group, obtained from image analysis, are reported in Table 1 and Supplementary Figure S1. In our previous studies [10,11], alveolar septal destruction/loss and airspace enlargement had been shown to become evident in these models by day 21 as established emphysema. Such alveolar structural abnormalities persisted after saline treatment: MLI and %DI values on day 35 were 89.6 ± 4.4 μm and 12.1 ± 1.5% in the CSE-induced model, and 82.8 ± 2.9 μm and 28.2 ± 7.3% in the SU5416-induced model, respectively, which were significantly greater than 53.6 ± 0.9 μm and 4.2 ± 0.3% in healthy controls. In contrast, after CDSO3 treatment at 60 μg/kg, MLI and %DI values were 63.4 ± 1.1 μm and 5.9 ± 0.7% in the CSE-induced model, and 62.9 ± 5.2 μm and 10.2 ± 2.6% in the SU5416-induced model, corresponding to 68–79% recovery. However, CDSO3 pre-mixed with FeSO_4_ showed only by 21–38% recovery, again supporting a mechanism mediated by Fe^2+^ chelation. In healthy rats, MLI and %DI values remained normal following CDSO3 treatment.

### 3.5. Stimulation of Cell Proliferation in Rats with Established Emphysema

**Figure 6:**
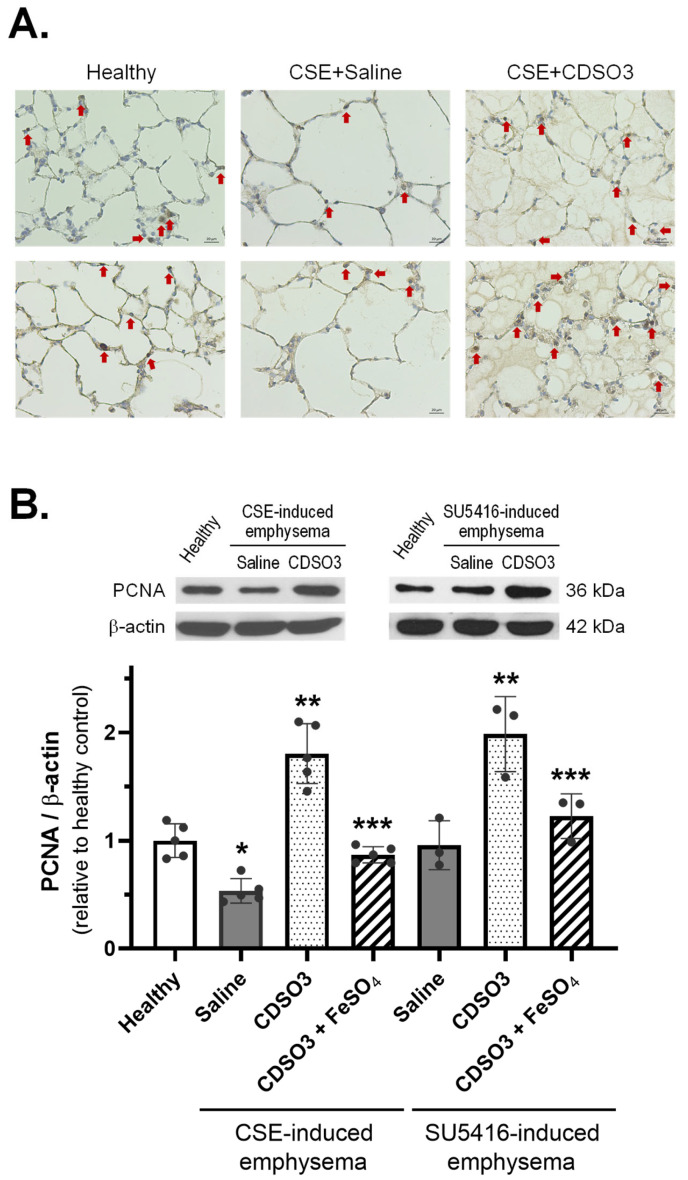
Lung PCNA expression in emphysematous rats treated with saline or CDSO3 at 60 μg/kg, compared with healthy rats. (**A**) Representative images of alveolar airspaces in CSE-induced emphysema (scale bar = 20 μm). Red arrows indicate PCNA-positive cells. (**B**) Western blot quantification of PCNA in CSE- and SU5416-induced emphysematous rats. CDSO3 at 60 μg/kg was also tested after pre-mixing with 10 mM FeSO4. Data: mean ± SD (n = 5 or 3). Dots: individual animal data. * *p* < 0.05 vs. healthy rats; ** *p* < 0.05 vs. saline-treated emphysematous rats; *** *p* < 0.05 vs. CDSO3-treated emphysematous rats.

As shown in Figure 6A, immunohistochemical PCNA expression was sparse in the alveolar airspaces of CSE-induced emphysematous rats following saline treatment but increased following CDSO3 (60 μg/kg) treatment. Western blotting, shown in Figure 6B, confirmed that in CSE-induced emphysema, lung PCNA levels were 47% lower after saline treatment, whereas CDSO3 not only restored but also elevated PCNA expression to 1.8-fold above healthy levels. In SU5416-induced emphysema, PCNA expression remained unchanged by saline treatment but again increased significantly by 2.0-fold with CDSO3. However, in both models, CDSO3 pre-mixed with excess Fe^2+^ resulted in only minor and insignificant increases in PCNA levels. Hence, these 1.8–2.0-fold increases in lung cell proliferation beyond healthy levels likely contributed to the restoration of alveolar structure and exercise endurance (Figure 4 and Figure 5; Table 1). These findings are also consistent with the in vitro cell-based results (Figure 2 and Figure 3), further supporting a Fe^2+^ chelation-mediated mechanism of action.

### 3.6. Suppression of Apoptotic Cell Death in Rats with Established Emphysema

**Figure 7:**
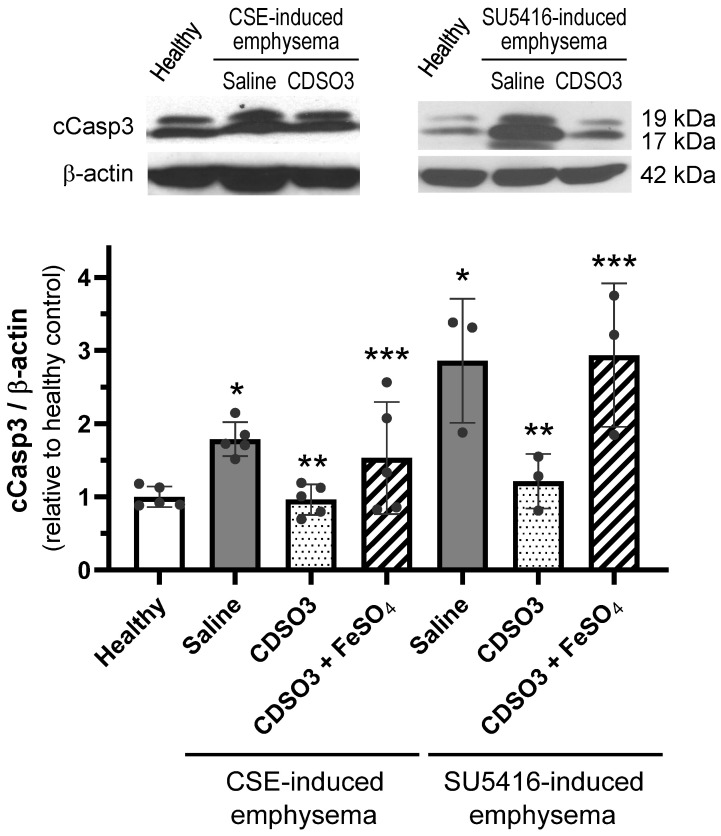
Lung cCasp3 expression in CSE- and SU5416-induced emphysematous rats treated with saline or CDSO3 at 60 μg/kg, compared with healthy rats, measured by Western blotting. CDSO3 at 60 μg/kg was also tested after pre-mixing with 10 mM FeSO_4_. Data: mean ± SD (n = 5 or 3). Dots: individual animal data. * *p* < 0.05 vs. healthy rats; ** *p* < 0.05 vs. saline-treated emphysematous rats; *** *p* < 0.05 vs. CDSO3-treated emphysematous rats.

Our previous study [10] demonstrated that CDSO3 inhibited in vitro lung cell death induced by various emphysematous insults. Thus, lung expression of cCasp3, a marker of apoptotic cell death, was evaluated in CSE- and SU5416-induced emphysematous rats, compared to healthy rats (Figure 7). After saline treatment, cCasp3 expression was significantly elevated by 1.7-fold in CSE-induced emphysema and 2.9-fold in SU5416-induced emphysema, suggesting that induced apoptosis contributed to alveolar septal destruction/loss (shown in Figure 5). In contrast, treatment with CDSO3 (60 μg/kg) almost completely suppressed these increases, restoring cCasp3 levels to those of healthy lungs (Figure 7). This suggests that the alveolar structural recovery with CDSO3 likely resulted not only from enhanced lung cell proliferation/migration (Figure 6) but also from inhibition of apoptosis (Figure 7). However, CDSO3 pre-mixed with FeSO_4_ was not able to inhibit cCasp3 induction, again supporting a Fe^2+^ chelation-dependent mechanism of its survival activity.

### 3.7. Recovery/Stimulation of HIF-1α and VEGF in CSE-Induced Emphysema

**Figure 8:**
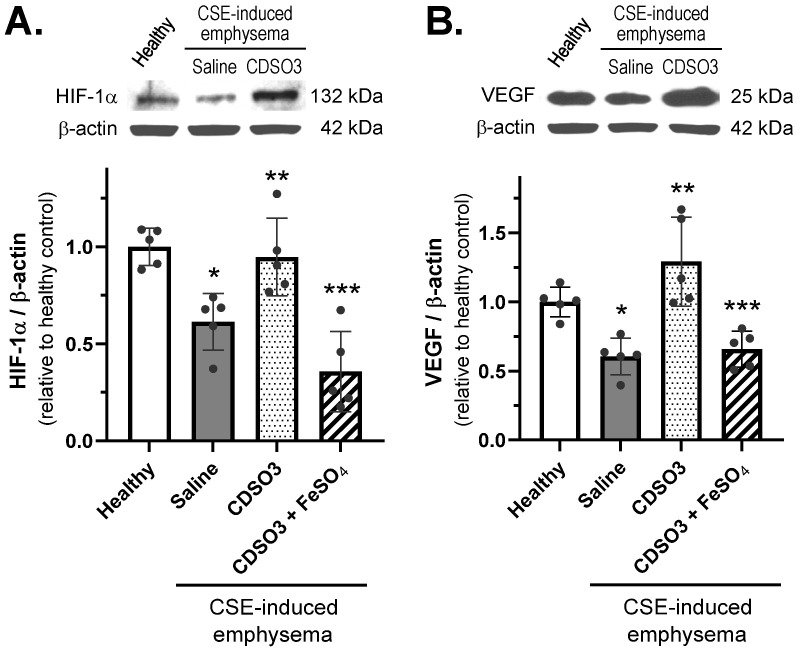
Lung tissue expression of (**A**) HIF-1α and (**B**) VEGF in CSE-induced emphysematous rats treated with saline or CDSO3 at 60 μg/kg, compared with healthy rats, measured by Western blotting. CDSO3 at 60 μg/kg was also tested after pre-mixing with 10 mM FeSO_4_. Data: mean ± SD (n = 5 or 3). Dots: individual animal data. * *p* < 0.05 vs. healthy rats; ** *p* < 0.05 vs. saline-treated emphysematous rats; *** *p* < 0.05 vs. CDSO3-treated emphysematous rats.

CDSO3 effectively restored lung expression of (A) HIF-1α and (B) VEGF in CSE-induced emphysematous rats (Figure 8). In saline-treated animals, HIF-1α and VEGF levels were significantly reduced by 38% and 41%, respectively, indicating “HIF-1α and VEGF deficiency” in this disease animal model, consistent with observations in emphysema and COPD patients [13]. In contrast, treatment with CDSO3 (60 μg/kg) restored HIF-1α to near-healthy levels and increased VEGF expression by 26% above healthy lungs. However, CDSO3 pre-mixed with FeSO_4_ again failed to restore HIF-1α or stimulate VEGF expression in these emphysematous lungs. These results suggest that CDSO3 reverses established emphysema, at least in part, through Fe^2+^ chelation-dependent stabilization of HIF-1α and subsequent VEGF upregulation.

## 4. Discussion

We demonstrated in this study that CDSO3 potently promoted proliferation and migration of lung epithelial and endothelial cells via a mechanism involving Fe^2+^ chelation and HIF-1α (Figure 2 and Figure 3). Local lung administration of CDSO3 at 60 μg/kg restored alveolar structure and improved exercise endurance by 68–79% in two rat models of established emphysema (Figure 4 and Figure 5; Table 1). Consistent with the in vitro results (Figure 2), CDSO3 treatment elevated lung PCNA levels by 1.8-fold relative to healthy lungs (Figure 6), suggesting enhanced cell proliferation. Lung cCasp3 levels, which were elevated 2–3-fold in emphysematous lungs, also returned to normal after CDSO3 treatment (Figure 7), as inhibition of apoptotic cell death, consistent with our previous in vitro findings [10]. These outcomes appear to result from HIF-1α stabilization and VEGF stimulation in the lungs (Figure 8). When Fe^2+^-chelated CDSO3 was administered, the restorative effects and HIF-1α and VEGF activation were all no longer observed (Figure 4, Figure 5, Figure 6, Figure 7 and Figure 8), further supporting the critical role of Fe^2+^ chelation in the observed CDSO3’s reversal efficacy. In these studies, body weight and lung weight did not differ between treatment groups. It is likely therefore that CDSO3 treatment did not produce overt adverse effects in these animal models.

A549 epithelial and HMVEC-L endothelial cells were used as in vitro models, since these two cell types form the alveolar septum and are destroyed and lost in emphysema. A549 cells are of tumor origin and thus may not fully represent primary alveolar epithelial physiology; however, normal alveolar epithelial cells are unavailable for in vitro use. A549 cells indeed retain progenitor-like type II epithelial cell properties, including proliferation, migration, and differentiation into type I epithelial cells (which cover > 95% of the alveolar surface), and respond to lung injury and damage [14]. These cells have also been used in studies of lung repair and VEGF-relating signaling as a reproducible and well-characterized model system [15,16]. The vascular endothelium is also essential for maintaining alveolar structure/function, and emphysema was linked to its injury [4,17]. Endothelial VEGF and its receptor expressions are reduced in the lungs of patients with emphysema/COPD, correlated with disease severity [18]. In animals, *systemic* infusion of healthy lung endothelial cells was shown to reverse established emphysema [18]. All these findings thus underscore the relevance of our in vitro epithelial and endothelial models for assessing CDSO3’s restorative potential. We then evaluated CDSO3 in two rat models of established emphysema induced by *systemic* administration of CSE and SU5416. Although chronic cigarette smoking is a primary risk factor, emphysema can still develop in ex-smokers long after cessation [19], suggesting the pivotal role of persistent *systemic* (and local) pathobiologic factors such as epigenetic alterations for disease progression. Emphysema also arises in genetic AATD, presumably due to reduced *systemic* antiprotease activity which somehow leads to local elastase overactivity in the lungs to cause alveolar destruction/loss [2,20]. These observations collectively imply that while emphysema manifests locally in the lungs, *systemic* factors significantly contribute to impaired alveolar structural maintenance. Even so, CDSO3 was capable of restoring alveolar structure and exercise endurance by 53–65% in a rat model of established emphysema *locally* induced with elastase and CSE, as shown in Supplementary S2 (Figures S2 and S3).

Our mechanistic rationale centered on HIF-1α/VEGF deficiency in emphysematous lungs, a proposed driver of alveolar structural destruction/loss [4,13]. HIF-1α is a key transcriptional regulator of VEGF, and its suppression leads to VEGF downregulation [21]. In emphysema/COPD patients, lung HIF-1α and VEGF expression levels are reduced and correlate with disease severity as well as histone deacetylase 2 levels implicating epigenetic impairment [13]. Excessive iron accumulation may also contribute, as reported in the lungs of cigarette smokers and emphysema/COPD patients [22]. Chronic inhalation of FeSO_4_ in coal dust was attributed as a cause of emphysema in coal miners, a major occupational lung disorder [23]. Mechanistically, Fe^2+^ activates HIF-degrading prolyl hydroxylases (HIF-PHDs), promoting HIF-1α degradation [24]. Even so, hypoxemia in emphysema/COPD might be expected to rather elevate HIF-1α; indeed, increased HIF-1α and VEGF expression has been reported in the lungs of smokers with COPD [25], highlighting the complexity of HIF-1α regulation. Nevertheless, our findings support a model in which HIF-1α/VEGF activation restores lung structure/function in emphysema.

CDSO3 is not the first molecule shown to reverse established emphysema in animal models; prior examples include simvastatin [26], all-trans retinoic acid [27], lecithinized superoxide dismutase [28], GHK tripeptide [29], hepatocyte growth factor [30], β1 integrin antibody [31], and recently, salvianolic acid B [11]. However, the mechanisms of action are largely undefined for most of these agents. Notably, simvastatin stimulates VEGF via HIF-1α upregulation [32], a mechanism parallel to that proposed for CDSO3, whereas salvianolic acid B activates VEGF through signal transducer and activator of transcription 3 (STAT3) signaling [10]. Both agents, like CDSO3, promote lung cell proliferation and migration and inhibit emphysematous cell death [10,26,32]. However, the degree of alveolar structural recovery for CDSO3 (68–79%) appears superior to that described for most other molecules. Moreover, the dual mechanism of Fe^2+^ chelation-mediated HIF-1α stabilization and multi-mechanistic anti-emphysematous activity [8,9,10] can distinguish CDSO3 from these previously reported molecules.

Our findings support the HIF-1α/VEGF activation-based strategy to restore alveolar structures and functions in HIF-1α/VEGF deficient emphysema. Likewise, HIF-1α/VEGF deficiency has also been implicated in bronchopulmonary dysplasia (BPD) in premature infants, in which incomplete lung development and emphysema-like airspace destruction are linked to elevated HIF-PHD expression [33,34]. Pharmacological HIF inhibition or VEGF suppression was shown to induce BPD-like airspace enlargement in neonatal animals, whereas HIF-PHD inhibition with FG-4095, HIF-1α gene transfer, or VEGF gene therapy promoted recovery from BPD-like lung defects [33,34,35]. Although the mechanisms underlying developmental defects in BPD likely differ from those responsible for alveolar destruction/loss in mature emphysema, these findings provide additional support for targeting HIF-1α and VEGF activation with CDSO3 to reverse emphysema.

Deferoxamine (DFO), an FDA-approved iron chelator, also stabilizes HIF-1α, stimulates VEGF, and promotes cell proliferation/migration, but only at ≥50 μM [36]. In contrast, another metal chelator, ethylenediaminetetraacetic acid (EDTA), lacks such activity. In our previous study [10], however, Fe^2+^ chelation potency was found to be comparable among CDSO3, DFO, and EDTA. Hence, it is the greater lipophilicity of CDSO3 that enables enhanced cellular penetration for intracellular Fe^2+^ chelation and potent reversal activity. Indeed, CDSO3 was effective at 60 μg/kg administered three times weekly via local lung delivery, whereas DFO required daily systemic injections of 150 mg/kg to improve alveolar development in a BPD model [37]. The larger molecular weight of CDSO3 (~3 kDa) likely also contributes to prolonged lung retention and efficacy due to slow diffusive lung absorption [38]. As a result, CDSO3 favorably retains in the lungs for a longer duration compared to DFO (561 Da).

## 5. Conclusions

CDSO3 enhanced lung cell proliferation and migration (Figure 2 and Figure 3) and reversed features of established emphysema in two rat models, restoring alveolar structure and exercise capacity by 68–79% via local lung delivery (Figure 4 and Figure 5; Table 1). These effects were associated with increased cell proliferation and reduced apoptosis and appear to involve Fe^2+^ chelation-mediated HIF-1α stabilization and VEGF stimulation (Figure 6, Figure 7 and Figure 8). Pre-chelation of CDSO3 with Fe^2+^ indeed abolished all these responses (Figure 2, Figure 3, Figure 4, Figure 5, Figure 6, Figure 7 and Figure 8). To date, no pharmacological agent has been shown to reverse emphysema in patients. Despite promising results in preclinical disease models, neither all-trans retinoic acid nor simvastatin has demonstrated clear benefits in clinical trials [39,40]. While the present findings are also limited to preclinical models and many further studies are still required, CDSO3 may represent a potential therapeutic candidate for local lung delivery (e.g., via inhalation), particularly by virtue of targeting a novel pathogenic mechanism, “HIF-1α/VEGF deficiency”, in addition to its previously demonstrated multi-mechanistic actions [8,9,10].

## Figures and Tables

**Table 1:** Mean linear intercept (MLI) and % destructive index (%DI) of alveolar airspace in different treatment groups of animals.

Group	Treatment	MLI [μm]	%DI [%]
Healthy	—	53.6 ± 0.9	4.2 ± 0.3
	CDSO3 60	58.2 ± 2.9	5.2 ± 1.8
CSE-induced emphysema	Saline	89.6 ± 4.4 *	12.1 ± 1.5 *
	CSDO3 10	70.0 ± 2.2 **	9.4 ± 1.8 **
	CDSO3 60	63.4 ± 1.1 **	5.9 ± 0.7 **
	CDSO3 60 + FeSO_4_	75.9 ± 1.5 ***	9.3 ± 1.1 ***
SU5416-induced emphysema	Saline	82.8 ± 2.9 *	28.2 ± 7.3 *
	CDSO3 60	62.9 ± 5.2 **	10.2 ± 2.6 **
	CDSO3 60 + FeSO_4_	74.3 ± 2.6 ***	23.1 ± 2.9 ***

CDSO3 10: CDSO3 at 10 μg/kg; CDSO3 60: CDSO3 at 60 μg/kg; CDSO3 60 + FeSO_4_: CDSO3 at 60 μg/kg pre-mixed with 10 mM FeSO_4_ Data: mean ± SD (n = 3–9); individual animal data are also shown in graphical format as Figure S1 in the Supplementary S1. * *p* < 0.05 vs. healthy control; ** *p* < 0.05 vs. the corresponding emphysema control treated with saline; and *** *p* < 0.05 vs. the corresponding emphysematous animals treated with CDSO3 at 60 μg/kg.

## Data Availability

The original contributions presented in this study are included in the article/Supplementary Material. Further inquiries can be directed to the corresponding author(s).

## Supplementary Materials

The following supporting information can be downloaded at: https://www.mdpi.com/article/10.3390/biology15070564/s1. Figure S1: Mean linear intercept (MLI) and % destructive index (%DI) of alveolar airspaces in different groups of animals. Bars: mean ± SD (n = 3–9). Dots: individual animal data. * *p* < 0.05 vs. healthy control; ** *p* < 0.05 vs. the corresponding emphysema control treated with saline; *** *p* < 0.05 vs. the corresponding emphysematous animals treated with CDSO3 at 60 μg/kg. Figure S2: Pre- and post-treatment treadmill exercise endurance times in HSE/CSE-induced emphysematous rats treated with saline or CDSO3 at 60 μg/kg, compared with healthy rats. On day 21, exercise endurance times significantly declined to 3–13 min in emphysema-induced animals. CDSO3 or saline was then administered to the lungs three times weekly for two weeks until day 35. Data: individual animal values. Horizontal bars: group means. *p* < 0.05 vs. no change (i.e., 0); NS: not significant. Figure S3: Representative alveolar airspace images for HSE/CSE-induced emphysematous rats treated with saline or CDSO3 at 60 μg/kg, compared with healthy rats.
