# Supplementary material for: Local Lung HIF-1α and VEGF Activation to Reverse Emphysema by a Sulfated Caffeic Acid Dehydropolymer
Source: Biology (Basel). 2026 Apr 1;15(7):564. doi: 10.3390/biology15070564 (PMC13071980; doi:10.3390/biology15070564)
Supplement: Supplementary file 1 [file biology-15-00564-s001.zip › biology-4147313-supplementary.pdf]

Supplemental Information

Local lung HIF-1α and VEGF activation to reverse emphysema by a sulfated caffeic acid dehydropolymer

Tien M. Truong[1], Meghan L. Thompson[2], Umesh R. Desai[2], and Masahiro Sakagami[1*]

[1]Department of Pharmaceutics, School of Pharmacy, Virginia Commonwealth University, 410 North 12th Street, Richmond, Virginia 23298, USA

[2]Department of Medicinal Chemistry, School of Pharmacy; and Center for Drug Discovery, Virginia Commonwealth University, 800 East Leigh Street, Richmond, Virginia 23219, USA

*To whom correspondence should be addressed.

Masahiro Sakagami, Ph.D.
Department of Pharmaceutics
School of Pharmacy
Virginia Commonwealth University
410 North 12th Street, P.O. Box 980533
Richmond, VA 23298-0533, USA

Phone: +1-804-314-4530
E-mail: msakagam@vcu.edu

ORCID: 0000-0002-1387-3009

S-1. Graphical representation of morphological airspace data reported in Table 1

The morphological airspace data (MLI and %DI) presented as numerical group means ± SD in Table 1 are also shown in Fig. S-1 as bar graphs with individual animal data points. Although this format improves data transparency, the graphs appear visually dense and make intergroup differences less discernible. Hence, the data are presented in tabular format in the main manuscript.

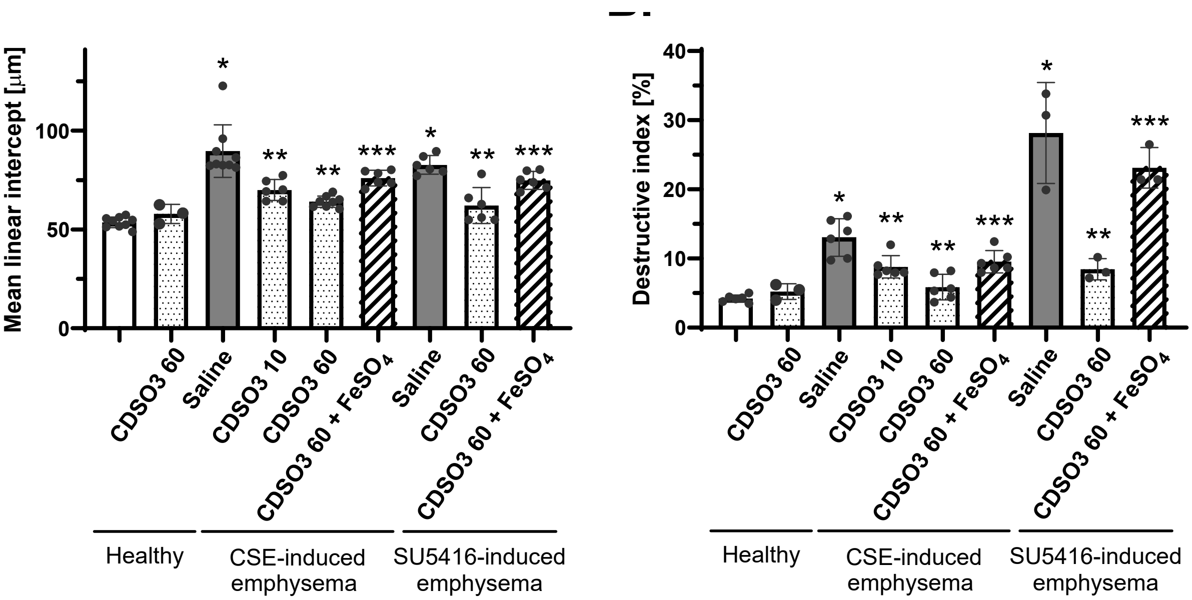

Figure S-1: Mean linear intercept (MLI) and % destructive index (%DI) of alveolar airspaces in different groups of animals. Bars: mean± SD (n=3-9). Dots: individual animal data. *$p<0.05$ *vs.* healthy control; **$p<0.05$ *vs.* the corresponding emphysema control treated with saline; ***$p<0.05$ *vs.* the corresponding emphysematous animals treated with CDSO3 at 60 μg/kg.

S-2. Recovery of exercise endurance and alveolar structure in rats with *established* emphysema induced by elastase and CSE

In addition to the CSE- and SU5416-induced emphysema models, CDSO3 was also evaluated in rats with *established* emphysema locally induced by elastase and cigarette

smoke extract (CSE) [9]. Rats received a single orotracheal (OT) spray (0.2 ml) instillation of human sputum elastase (HSE) and CSE on day 1 to induce emphysema. CSE solution was prepared by bubbling mainstream smoke from a 3R4F research cigarette through 3 ml of saline over ice using the smoking machine. The dosing solution was then prepared as an admixture of 0.05 ml HSE stock solution (8,750 U/ml) and 0.83 ml CSE solution. OT spray instillations were performed using a MicroSprayer under isoflurane anesthesia. Animals were then left untreated until day 21. On day 21, treadmill exercise endurance was assessed and found to have declined to 3~13 min, compared to ~45 min in healthy rats, confirming the development of *established* emphysema (Fig. S-2). From day 21, rats received OT spray instillations (0.1 ml) of either saline or CDSO3 at 60 μg/kg three times weekly for two weeks. On day 35, post-treatment exercise endurance times were measured on the treadmill, followed by euthanasia under urethane anesthesia.

Two-week treatment of CDSO3 at 60 μg/kg significantly improved treadmill exercise endurance in HSE/CSE-induced emphysematous rats (Fig. S-2). On day 21, endurance times were only 3~13 min, representing a marked 71~93% reduction relative to healthy controls (45.6±6.5 min) as *established* emphysema. This impaired exercise endurance remained unchanged in saline-treated rats, given post-treatment running times of 4~11 min on day 35. In contrast, CDSO3 treatment significantly improved post-treatment endurance, increasing running times by 53% to 27.6±9.1 min.

CDSO3 treatment (60 μg/kg) also restored alveolar airspace structure in HSE/CSE-induced emphysematous rats (Fig. S-3). In saline-treated rats, MLI and %DI values were 84.3±1.6 μm and 18.5±2.7%, respectively, significantly higher than those in healthy rats (53.6±0.9 μm and 4.2±0.3%), indicating persistent alveolar structural destruction/loss. By

contrast, CDSO3 treatment promoted structural recovery, as reflected by MLI and %DI values of 67.8±2.7 μm and 8.4±2.4%, corresponding to 53% and 71% recovery, respectively.

Together, these findings indicate that CDSO3 can also reverse *established* emphysema locally induced by HSE/CSE.

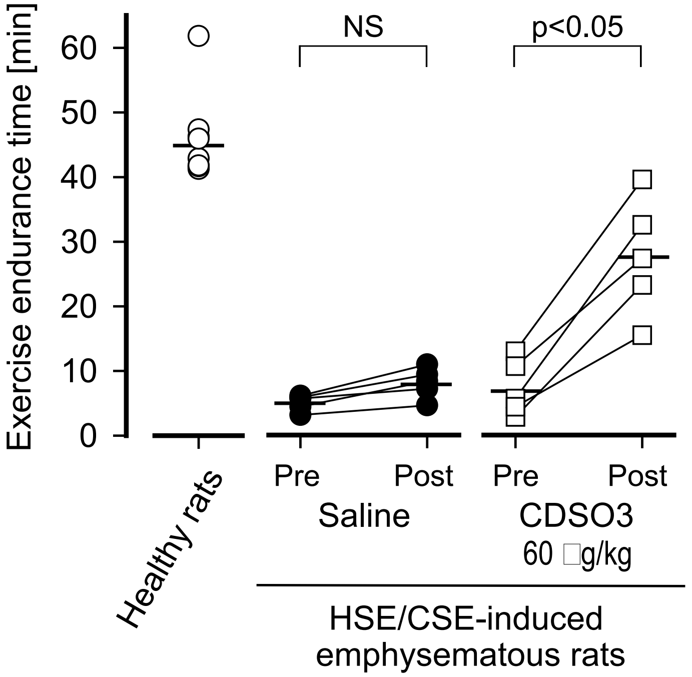

Figure S-2: Pre- and post-treatment treadmill exercise endurance times in HSE/CSE-induced emphysematous rats treated with saline or CDSO3 at 60 μg/kg, compared with healthy rats. On day 21, exercise endurance times significantly declined to 3-13 min in emphysema-induced animals. CDSO3 or saline was then administered to the lungs three times weekly for two weeks until day 35. Data: individual animal values. Horizontal bars: group means. p<0.05 *vs.* no change (i.e., 0); NS: not significant

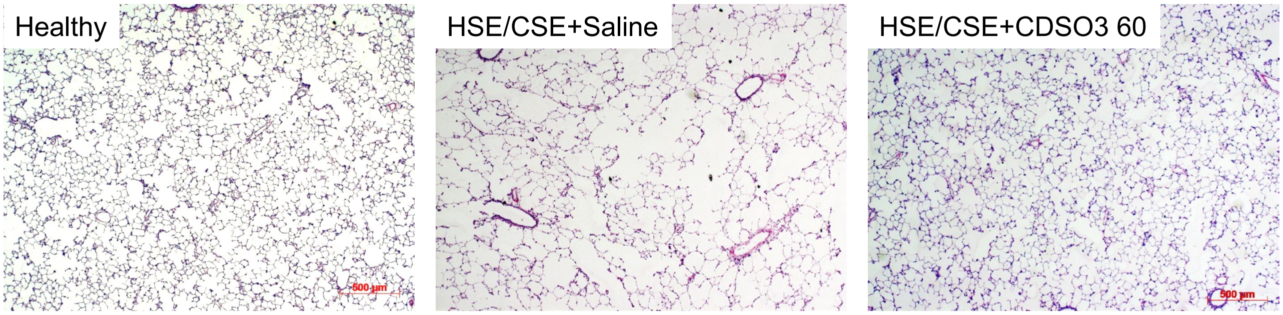

Figure S-3: Representative alveolar airspace images for HSE/CSE-induced emphysematous rats treated with saline or CDSO3 at 60 μg/kg, compared with healthy rats.
